# A fertility-sparing surgery in lymphoepithelioma-like carcinomas of the uterine cervix: A case report

**DOI:** 10.1097/MD.0000000000031579

**Published:** 2022-11-11

**Authors:** So Hee Kim, Hyung Joon Yoon, Nam Kyung Lee, Kyung Un Choi, Ki Hyung Kim, Dong Soo Suh

**Affiliations:** a Division of Gynecologic Oncology, Department of Obstetrics and Gynecology, Pusan National University School of Medicine, Busan, Republic of Korea; b Department of Radiology, Pusan National University School of Medicine, Busan, South Korea; c Department of Pathology, Pusan National University School of Medicine, Busan, South Korea.

**Keywords:** cervix uteri, fertility preservation, human papillomavirus type 16, squamous cell carcinoma, uterine cervical neoplasms

## Abstract

A poorly differentiated lymphoepithelioma-like carcinoma (LELC) of the cervix is an extremely rare presentation. We herein present an unusual case of LELC of the cervix, which was treated with radical trachelectomy for fertility preservation.

**Patient concerns:**

A 28-year-old female patient presented with a 1-month-history of post-coital vaginal bleeding, and a 2 cm tumor was found on gynecological sonography and magnetic resonance imaging.

**Diagnosis:**

The final pathological examination established a conclusive diagnosis of LELC of the cervix. After surgery, the patient was finally diagnosed as The International Federation of Gynecology and Obstetrics (FIGO) stage IB1 with no vaginal wall or parametrium infiltration.

**Interventions:**

Subsequently, a surgery was scheduled, and intraoperatively, we performed resection twice because of a frozen biopsy result that was resection margin-positive initially. As a result, further resection was performed, which was a 5mm thickness for each. Cisplatin adjuvant chemotherapy was administered 3 weeks after the operation to prevent recurrence.

**Outcomes:**

The patient has been followed for 1 year postoperatively, with an adjuvant treatment, with no evidence of tumor recurrence or metastasis.

**Conclusion:**

Based on this case, we highly recommend that operators should consider a deeper resection margin range than that visible on magnetic resonance imaging. More attention is needed to better understand the treatment method for LELC of the cervix. We also plan to closely monitor the patient’s prognosis and fertility, and to conduct additional studies.

## 1. Introduction

Cervical cancer is the third most common malignancy of the gynecologic tract, with an estimated 12,000 new cases diagnosed each year in the United States. In 2019, 58,000 new cases of cervical cancer were reported in Korea, accounting for 2.7% of total cancer incidence. Cervical cancer is classified histologically into numerous subtypes, with squamous cell carcinoma being the most common (65%–80%), followed by adenocarcinoma (15%–25%).^[1]^ Additionally, neuroendocrine tumors, adenosquamous carcinoma, carcinosarcoma, and non-epithelial neoplasms are less common histologies.

According to the World Health Organization’s histological categorization of tumors, lymphoepithelioma-like carcinoma (LELC) is an uncommon subtype that is best categorized as a variant of SCC in the cervix. In all patients with stage 1 cervical cancer, the incidence of LELC is approximately 3.3%.^[2]^ Although LELCs are frequently detected in the nasopharynx, they may also be found in the stomach, thymus, urinary bladder, lungs and salivary glands.^[3]^ Histologically, it is characterized by poorly differentiated tumor cells with abundant cytoplasm and intense chronic inflammatory infiltration.^[2]^

Various studies on the pathogenesis of LELC in Epstein-Barr virus (EBV) and Human Papillomavirus (HPV) infections have been published. EBV has been suggested as a possible causative factor of LELC, particularly in Asian women, but not in white women.^[4]^ The role of HPV in the pathogenesis of cervical LELC is unknown; the presence of HPV has been studied in only a small number of cases.

This report presents a case of LELC in a relatively young Korean woman who underwent radical trachelectomy for fertility preservation. This patient showed a HPV 16 positivity but absence of EBV.

## 2. Case presentation

A 28-year-old Korean woman, gravida 0, was referred to Pusan National University’s Department of Obstetrics and Gynecology. She visited a gynecologist of local medical center because of post-coital bleeding that persisted for a month. Her most recent gynecological examination and cervical smear were both normal 1 year ago. Cytology and histology tests performed at a local medical center revealed squamous cell carcinoma. A HPV DNA chip test detected HPV-16. Additional gynecological ultrasonography revealed a tumor in the cervix measuring 2 cm in diameter.

**Figure 1.**
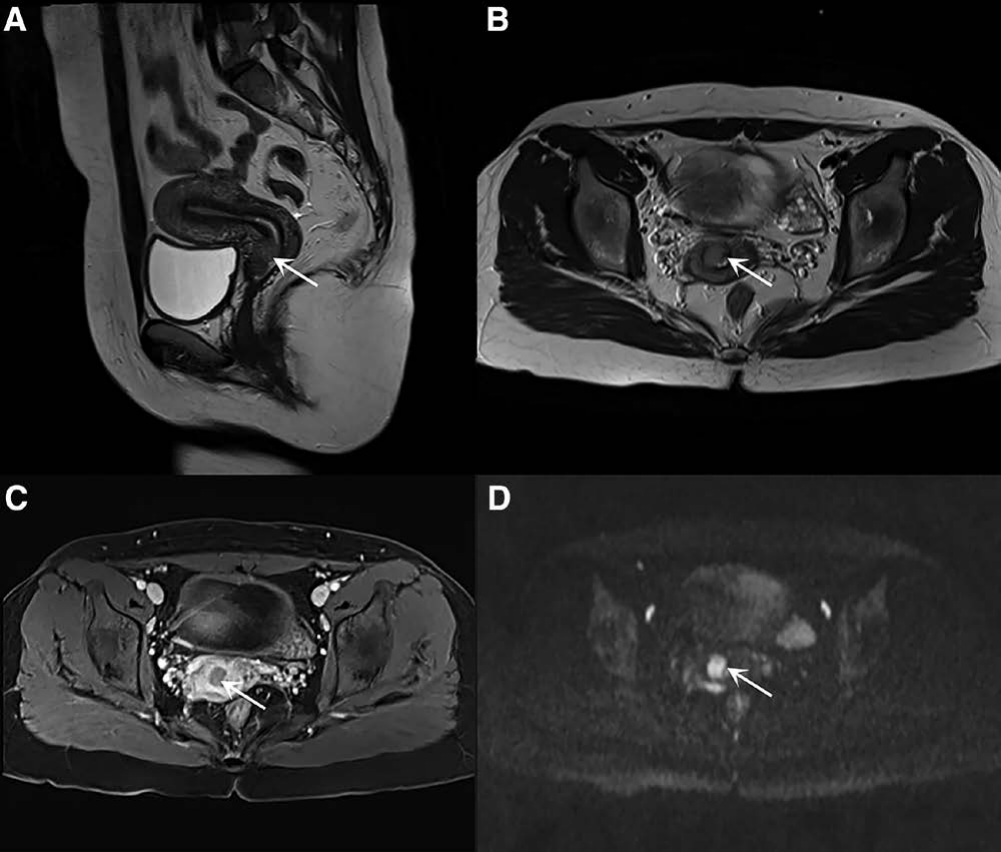
MRI of lymphoepithelioma-like carcinomas (LELCs) of the cervix (clock-wise A, B, C, D) (A, B) Sagittal (A) and axial (B) T2-weighted images show a focal mass (arrow) in the anterior lip of the exocervix. (C) Contrast-enhanced axial T1-weighted image shows a hypovascular cervical mass (arrow). (D) Diffusion-weighted image at b = 1000 s/mm^2^ shows hyperintensity (arrow) in the cervical mass. MRI = magnetic resonance imaging.

She was admitted to Pusan National University for further evaluation, which included a hematological profile, magnetic resonance imaging (MRI), and positron emission tomography. Her hematological profile was normal, and her serum squamous cell carcinoma antigen level (1.98 ng/mL) was also within normal limits. MRI and positron emission tomography revealed a 1.7 cm tumor in the anterior lip of the cervix (Fig. 1A–D) and a 2.2 cm hemorrhagic cyst in the right ovary, and no distant metastatic lesions or enlarged lymph nodes.

We determined that the patient’s radiology stage was FIGO (The International Federation of Gynecology and Obstetrics) IB1, and decided to perform trachelectomy rather than radical hysterectomy to preserve the patient’s fertility. Open radical trachelectomy and bilateral pelvic lymphadenectomy were performed in March 2021. During the operation, we performed resection twice because of a frozen biopsy result that was resection margin-positive initially. Then, we performed endocervical curettage for frozen biopsy. A frozen biopsy showed suspicion of endo-margin positive and loop electrosurgical excision was done for endocervix resection followed by abdominal cerclage.

**Figure 2.**
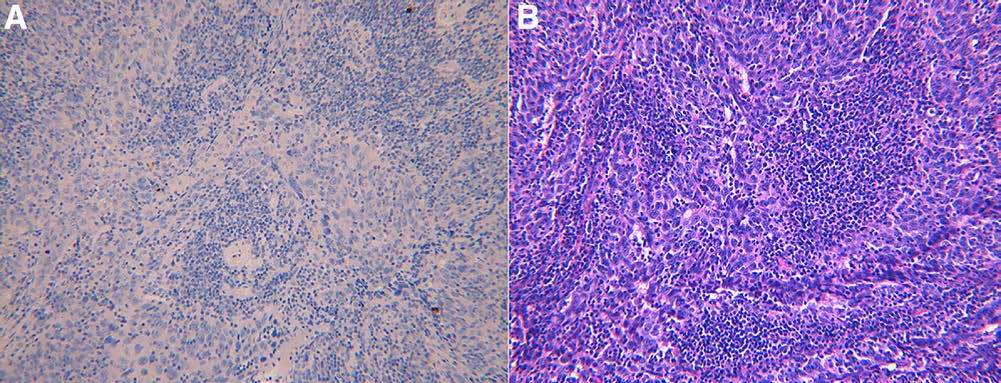
Microscopic findings. (A) [H&E × 200] The tumor shows poorly differentiated epithelioid cells with marked lymphoid cell infiltration. (B) [EBVISH × 200] Negative result of in situ hybridization for Epstein-Barr virus-encoded early RNA on formalin-fixed paraffin embedded tissue.

After surgery, the patient was finally diagnosed as FIGO stage IB1 with no vaginal wall or parametrium infiltration, and pathological examination revealed some tumor parts with extensive lymphocyte infiltration and lesions that resemble lymphoma and the immunostaining was positive for p53, diffuse positive p63, p16, and pan-cytokeratin. These findings are consistent with LELC of cervix. There was no evidence of lymphatic invasion (Fig. 2A) or lymph node metastasis. Additionally, in situ hybridization to EBV encoded early RNA (Fig. 2B) was performed on formalin-fixed paraffin-embedded tissue to investigate the association between EBV and LELC. However, the result was negative.

The patient’s postoperative course was uneventful. Cisplatin adjuvant chemotherapy was administered 3 weeks after the operation to prevent recurrence. The patient was followed for 1 year after 6 cycles of cisplatin 80 mg chemotherapy with no evidence of recurrence and other complications.

## 3. Discussion

LELC is an uncommon subtype of squamous cell carcinoma. Previous studies on LELC of the cervix between 1997 and 2017 are listed in Table 1. Histologically, it was identified by nests of undifferentiated epithelial cells that grew in a syncytial pattern and were infiltrated by severe lymphocyte infiltrates.^[15]^ LELCs have been included in prior reviews of “inflammatory” cervical neoplasms.^[16]^ This microscopic characteristic can help in differentiating LELC of the cervix.

**Table 1:** Reported cases of LELC of cervix in the literature.

Author	Country	Case no.	Age (yr) (or median)	EBV	HPV	FIGO stage	Treatment	Adjuvant treatment
Tseng et al^[5]^	Taiwan	15	56 (37–72)	Negative	16	IB(9)	RH, BPLND	None
						IIA1(2)	Radiotherapy	
						IIB(4)		
Takai et al^[6]^	Japan	3	41	Negative	Negative	IB1	RH, BPLND	No report
			69	Negative	Negative	IIA	RH, BPLND	No report
			44	Negative	Negative	IB1	RH, BPLND	No report
Saroha et al^[7]^	India	2	40	Not done	Not done	IB1	RH, BPLND, PALND	No report
			43	Not done	Not done	IB1	RH, BSO, BPLND, PALND	No report
Bais et al^[2]^	Netherlands	1	44	Negative	16, 45	IB1	RH, BPLND	None
Table 1. Reported cases of LELC of cervix in the literature								
Martorell et al^[8]^	Spain	4	74	Negative	Negative	IB1	RH, BPLND	No report
			58	Negative	Negative	IB1	RH, BPLND	No report
			77	Negative	Negative	II	RH, BPLND	No report
			67	Negative	Negative	IB1	RH, BPLND	No report
Noel et al^[4]^	Belgium	2	56	Negative	16	IB1	RH, BPLND	No report
			53	Negative	18	IB1	RH, BPLND	No report
Lopez-Rios et al^[9]^	Spain	1	44	Positive	Not done	IB1	RH, BPLND	No report
Saylam et al^[10]^	Belgium	1	72	Not done	18	IB1	RH, BPLND	None
Takebayashi et al^[11]^	Japan	1	45	Negative	Not done	IB3	RH	Radiotherapy
Table 1. Reported cases of LELC of cervix in the literature								
Kaul et al^[12]^	India	1	42	Not done	Not done	IB1	RH, BPLND	No report
Kim et al^[13]^	Korea	1	57	Negative	Negative	IB1	RH, BSO, BPLND, PALND, appendectomy	Paclitaxel + cisplatin
Ki et al^[14]^	Korea	1	63	Positive	16	IB1	RH, BSO, BPLND, appendectomy	None
Yoo et al^[15]^	Korea	1	55	Not done	Not done	IB1	RH, BSO, BPLND	None
Yun et al^[15]^	Korea	1	45	Negative	Negative	IB1	RH, BPLND	None

BPLND = bilateral pelvic lymph node dissection, BSO: Bilateral salpingo-oophorectomy, EBV = Epstein-Barr virus, FIGO stage = 2018 FIGO Staging System for Uterine Cervical Cancer, HPV = human papilloma virus, PALND = para-aortic lymph node dissection, RH = radical hysterectomy.^[19]^

The pathogenesis of LELC is unclear; however, it is assumed that either EBV or HPV plays an important role as a causative factor.^[17]^ EBV is not directly involved in squamous cell malignant transformation, but may have an effect on the inflammatory component. Tseng et al^[5]^ reported that 73.3% (11/15) of Asian women with LELC of the cervix were positive for EBV antibodies.^[15]^ However, there are no definite findings that EBV acts as the main factor in cervical cancer in Caucasians.^[9]^ It can be assumed that race and ethnicity can influence the role of EBV gene as an oncogenic factor.^[5]^ With regards to HPV, an association with HPV infection has also been postulated. HPV infection has been identified as a causative factor in the pathogenesis of cervical cancer. Despite this assumption, it is difficult to find a specific association between EBV and HPV positivity in Asian cases (Table 1). In this case, in situ hybridization for EBV-encoded early RNAs was performed, and there was no EBV infection, but HPV 16 was positive.

Patients with LELC had an average age of 49.6 years. Fourteen patients (82.4%) were classified as FIGO IBI, while 3 patients (17.6 %) were classified as FIGO IB2. Three patients (17.6 %) had metastatic lymph nodes, thirteen (76.5 %) had non-metastatic lymph nodes, and 1 patient had been unknown.^[18]^ The incidence in Asia is higher than that in Western countries.^[8]^ Our case was a 28 years old woman, which was younger than the mean age.

There have been some cases and original articles on LELC of the cervix. Most prior cases focused on the diagnosis and case itself. There was not much focus on the treatment of LELC. When it comes to treatment, while radiation therapy can be used at any stage of disease, surgery is only recommended for patients with stage I to IIA disease. LELC has a better prognosis than other types of cervical cancer.^[2]^ Van Nagell et al^[19]^ also reported that LELC of the cervix had lower lymph node metastasis and recurrence rate. Radical hysterectomy is a surgical procedure that is commonly used to treat LELC of the uterus. In this case, we performed radical trachelectomy to preserve fertility. This is the first case of LELC treated with radical trachelectomy in Korea.

Before surgery, we determined the resection margin using the MRI results. During the operation, we performed resection twice because of a frozen biopsy result, which was endo-margin positive at first. The second endocervical resection was performed using the loop electrosurgical excision. Further resection was a 5 mm thickness for each. Therefore, we resected the cancerous lesion at 1cm above from its suspected margin according to its MRI. Based on this experience, we highly recommend that the operator should consider the margin range deeper than that seen on MRI. It’s not certain whether it was just this patient’s physical feature or LELC of the cervix itself. Since LELC of the cervix is a rare disease, there are still many issues to be considered. More attention is needed to better understand the treatment method for LELC of the cervix. We also plan to closely monitor the patient’s prognosis and fertility, and to conduct additional studies.

This patient has agreed an informed consent before this case report was written.

## Author contributions

**Conceptualization:** Dong Soo Suh.

**Supervision:** Ki Hyung Kim, Dong Soo Suh.

**Validation:** Kyung Un Choi.

**Visualization:** Nam Kyung Lee.

**Writing – original draft:** So Hee Kim.

**Writing – review & editing:** Hyung Joon Yoon.
